# The impact of social media use on student engagement and acculturative stress among international students in China

**DOI:** 10.1371/journal.pone.0284185

**Published:** 2023-04-14

**Authors:** Blessing Dwumah Manu, Feng Ying, Daniel Oduro, John Antwi, Robert Yakubu Adjuik

**Affiliations:** 1 School of Management Science and Engineering, Jiangsu University, Zhenjiang, Jiangsu, P.R. China; 2 Universitat Autonoma De Barcelona, Barcelona, Spain; 3 University for Development Studies, Tamale, Ghana; 4 Simon Diedong Dombo University of Business and integrated Development Studies, Bamahu, Ghana; Babes-Bolyai University, Cluj-Napoca, ROMANIA

## Abstract

Despite the widespread use of modern social media, relatively less is known about the impact of social media on the acculturation processes of international students in China and their engagement in school activities. Accordingly, this research intends to assess the influence of social media usage while answering questions such as how using social media can improve international students’ acculturation process from both psychological/mental and behavioural standpoints, as well as whether international students’ acculturation promotes students’ engagement in school activities, among other questions. The role of self-identification in mediating the connection between social media usage and international students’ acculturation is also investigated. Primary data were gathered from 354 international students studying at various universities around China. The results show that international students use of social media improves their acculturation process and engagement in school activities through information sharing, establishing contacts, and entertainment. The study’s limitations and future directions are also highlighted.

## 1. Introduction

**Fig 1:**
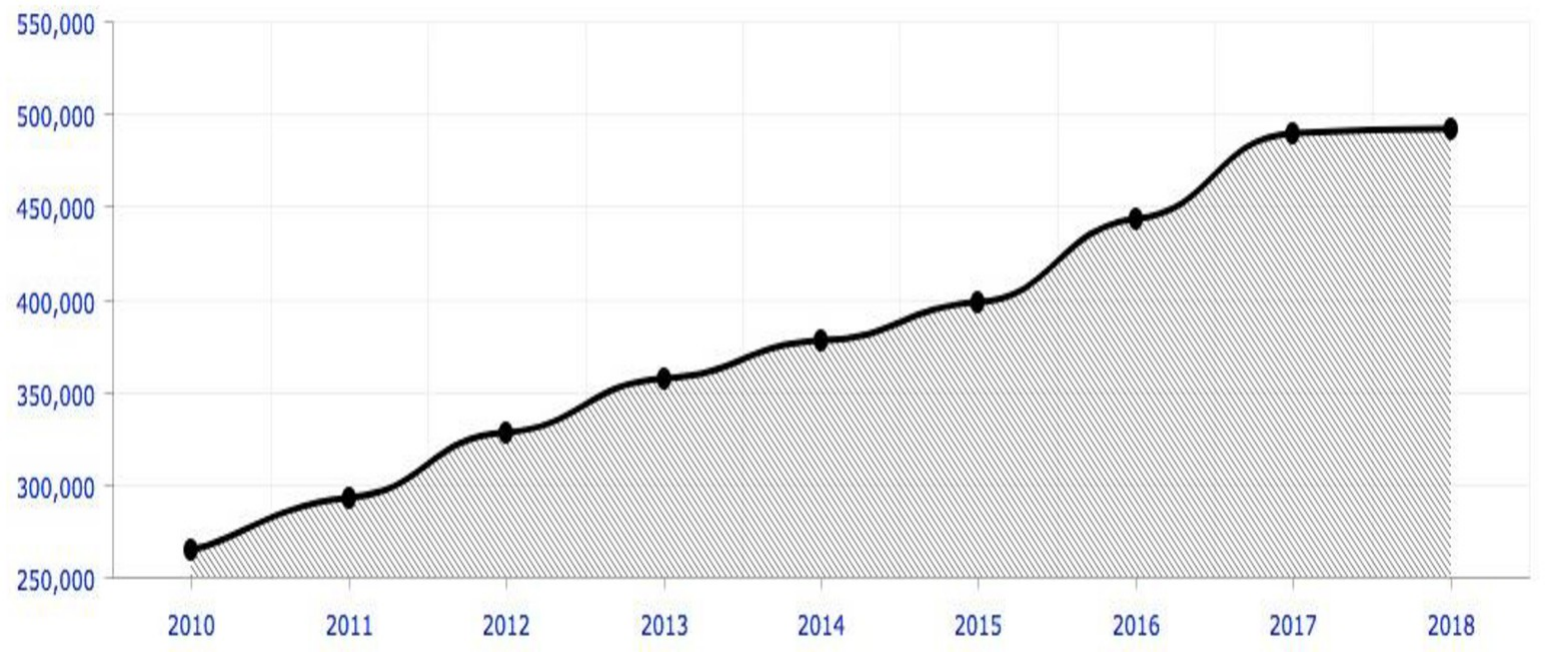
Number of international students in Chinese universities (2010–2018). Source: Chinese Ministry of Education (MoE) Statistics Yearbook 2010–2018.

Globalization, internationalization, and human mobility have increased the number of persons considering education abroad [1]. Studies show that the number of international students living and learning abroad has risen from 800,000 in 1975 to 4.6 million in 2015, and it is predictable to reach 7.2 million by 2025 (OECD, 2017, 2013). While the United States (US), the United Kingdom (UK), Australia, France, Canada, Russia, and Germany have always been the favourite destination for students seeking higher education overseas, non-English-speaking countries, like, Japan, South Korea, India and China have also risen as desirable destinations for higher education [2]. Particularly, with about 500,000 overseas students enrolled in 2020, the rapidly expanding Chinese economy has developed its higher education into an "*international education center*" (See Fig 1). China has moved from being the top sender of international students to one of the top ten host countries of international students. Since 2014, it has been the third-largest host country after the US and the UK [3].

Most international students studying abroad are engaged in various forms of social media platforms. Though the use of social media platform comes with positive effects such as creating and sharing information with others, examples include sites like Facebook, Instagram, Snapchat and Twitter, which allows us to connect and communicate with others, share a common challenge/interest, Access to Information, allows us to inspire others to do things, Furthermore, social media can increase voter participation as well. Besides, the ugly part of social media there are tons of unnecessary information shared by people and also bullying and harassment on social media has been increased. People can make brutal and negative comments about anything and anyone. Social media has good, bad and ugly impacts on our culture. As hypothesized, a separation in language preferences on social media has a significant and positive effect on enculturation and a negative effect on acculturation, suggesting that international students’ immigrants’ preferences for their heritage languages online lead to the maintenance of heritage cultures in offline contexts. There is a perceived discrimination and ethnic identity crisis including other risk factors in the process of acculturation in the use of social media platforms. Hence, social media use can also negatively affect students and teens, distracting them, disrupting their sleep, and exposing them to bullying, rumour spreading, unrealistic views of other people’s lives and peer pressure. The risk factors are many, hence an investigation to ascertain the impact of social media. This topic is wealth research because it has a role to play in international students’ life and acculturation in China.

In line with the above, international students contribute significantly to the host country’s economy and cultural diversity, they frequently struggle to adapt to a new culture, resulting in problems and stress during acculturation, which sometimes interferes with their academic performance [4]. Though social media comes along with many advantages, social media also has several disadvantages that in certain situations limit students’ concentration and academic progress in school. Social media can be a distraction for some graduate students. Students may be distracted from their school work and the teachers will have no option of knowing which student pays attention during lecture sessions. Research on acculturation indicates that adjusting to a new culture can be challenging [5]. Generally, people experience adverse feelings such as anxiety, isolation, depression, and other negative emotions [6]. However, as internet service and modern telecommunication technology, especially the use of social media, has increased over the past few decades, international students’ acculturation experiences and cognitive abilities to integrate into other communities have improved [4]. According to [5] social media contains technical and social features that allow international students to adjust quickly to the adaptation process while maintaining ties with their home country.

Consequently, previous empirical studies in the area of acculturation have consistently found that international students are more likely to use social media to expand their social networks, learn about the host country’s culture, form friendships with friends and family, and fulfil various needs in a non-native environment while quickly adapting to their new surroundings [5]. For instance, [7] surveyed 283 Korean and Chinese college students in the U.S. to assess their acculturative stress. They demonstrated that international students use social media to post writings and photos about their new lives in the host country, allowing them to maintain contact with important friends back home and thus improve their acculturation process. [8] use 146 international students in Argentina to show that social media and video chat could help international students find people with similar interests, make new friends in a new place, and feel less stressed about adapting to a new culture. [9] explore the role of major US social media in Chinese students’ acculturation and show that social media contributes to higher acculturation and better adaptation. [10] explore the role of social media on international students enrolling in South Korean universities and find that the use of social media helps to reduce international students’ acculturative stress and to enhance their satisfaction with Korean life. [6] survey international students at a large American university and discover that social media was associated with less stress from cultural adaptation because social support from the host country was greater. More recently, [11] examine how 27 transient international students in the UK use social media to negotiate and control their identities. The study shows that social media can help people maintain their home culture, adapt to the host culture, and integrate two cultures. Others have discovered that social media can help international students socially adapt to educational environments in other countries. By administering an online questionnaire to a group of 120 Chinese international students, [12] discover that those who utilize social media more frequently demonstrate greater social and academic adaptation to the foreign culture. Similarly, [4] investigate the psychological and behavioural effects of social media on the acculturation processes of Chinese students studying in the United Kingdom. Their findings indicate that the psychological acculturation of Chinese international students to the host culture is unrelated to their academic performance.

We define social media as an internet-based application that allows users to create a public or semi-public profile to interact, share, and exchange information and ideas with others in online communities and networks. Researchers have increasingly examined the relationship between international students’ media usage and their cross-cultural adaption in recent years, there have been relatively few studies on this topic in China. The majority of previous studies only concentrated on Chinese students’ experiences in US and UK universities and how their expectations were met or not. Perhaps, a surge in the number of Chinese students in US and UK universities may have increased the scholarly study on Chinese students. According to the International Education Exchange Open Doors report published by the Institute of International Education (2015), China accounts for roughly 31% of international students in the US. China also offers the UK, Australia, Canada, Japan, and New Zealand the largest number of international students. However, as indicated earlier, China, which once sent many of its students abroad, is progressively becoming a recipient of international students to boost inward student mobility [13].

International students in other nations may have similar experiences in host cultures, and certain cultural customs in China can make adaptation difficult for international students [14]. Despite historical and present influences from the West, China’s socio-cultural structure is generally different from the Western world and even its nearest neighbours. Chinese educators are aware of these disparities and are attempting to assist international students to adapt to Chinese sociocultural and educational practices [15]. Even though various aspects of international student adaptation tactics, particularly social media usage, have been proposed to increase acculturation and student engagement in China, there is no study investigating this issue [16]. Moreover, China uses a different set of social networking sites and apps than the rest of the world, and more importantly western social media platforms like Facebook, Twitter, Instagram, YouTube, and WhatsApp are blocked in China. To maintain the connection to these western social networks, a Virtual Private Network (VPN) is required to access the restricted Western social media platforms; however, VPN use is not always possible in China. Thus, the extent to which social media improves international students’ adaptation to Chinese culture and, ultimately, their school activities is questionable and remains unclear in international academic circles. Hence, this necessitates scholarly research on the effects of social media on international students’ acculturation and its outcomes on their university experience.

Building upon the foregoing discussions, the current study uses a quantitative research design to increase our present understanding of international students’ acculturation by (a) examining the impact of social media use on international students in China acculturation processes in terms of psychological (mental) and behavioural dimensions, and b) exploring the link between acculturation and student engagement among overseas students in China. Ethnic self-identification is employed as a moderator to investigate the intermediation effect of social media use on acculturation among international students. Ethnic self-identification is crucial to the ideological and behavioural changes during an individual acculturation process. Ethnic self-identification is defined by how tenaciously and forcefully they keep their ethnicity and cultural characteristics when engaging with people from various ethnic and cultural backgrounds.

The rest of the paper follows this particular order. Section 2 discusses the literature review and hypotheses. Section 3 highlights the methods and materials. Section 4 provides the results, and the section discusses the outcomes. Finally, section 5 closes the research with policy recommendations, limitations and further research.

## 2. Literature review

### 2.1 Acculturation and social media usage

Berry (2005) refers to acculturation as “*the dual process of cultural and psychological change that takes place as a result of contact between two or more cultural groups and their individual members*” (p.698). In other words, acculturation happens as people from various cultures interact on a daily basis, leading to changes in their mental (or psychological) well-being and behaviour as a whole [17]. Mental acculturation is changed in the psycho-cultural orientations of individuals [18]. While behavioural acculturation consists of multi-faceted languages, such as media uses, religion and food consumption [19, 20].

Like any other immigrant, previous studies have revealed that international students may find it hard to adjust to a new cultural setting and may suffer elevated stress levels due to the supposed cultural barrier. International students could face cultural "shock or stress" upon arrival in a new host environment, primarily impacting their mental well-being [21]. It is possible that they will experience significant cultural and ethnic differences between their own country and the host country [19]. They may struggle to communicate and study in different languages and cultures.

However, given the rapid advancement in technology, there is wide use of various social media platforms such as Facebook, WhatsApp, WeChat, etc., mainly among young people [22]. Research indicates that these social media platforms enable international students to connect with family, friends and coworkers almost anytime and from any location expediting their acculturation processes. As a result, scholars have attempted to study the link between social media use and acculturation from multiple perspectives. Many of these investigate whether home or host social media affect the acculturation process [23]. According to these studies, using media from the host country can help acculturation by offering access to mainstream culture, whereas using media from the home country can obstruct the process by limiting opportunities for cross-cultural communication. Cemalcilar et al. (2005), for instance, stated that using social media from the home country is linked to a greater degree of support for ethnic identity while using host countries’ social media is linked to stronger social and cultural adaption to the host country. Similarly, in the United States, [24] show that media consumption in the host nation correlates positively with social interaction, English proficiency and awareness of the host culture. More recently, [23] found that among 121 Chinese students in New Zealand, those who used host social media more frequently were more likely to identify with New Zealand.

**Fig 2:**
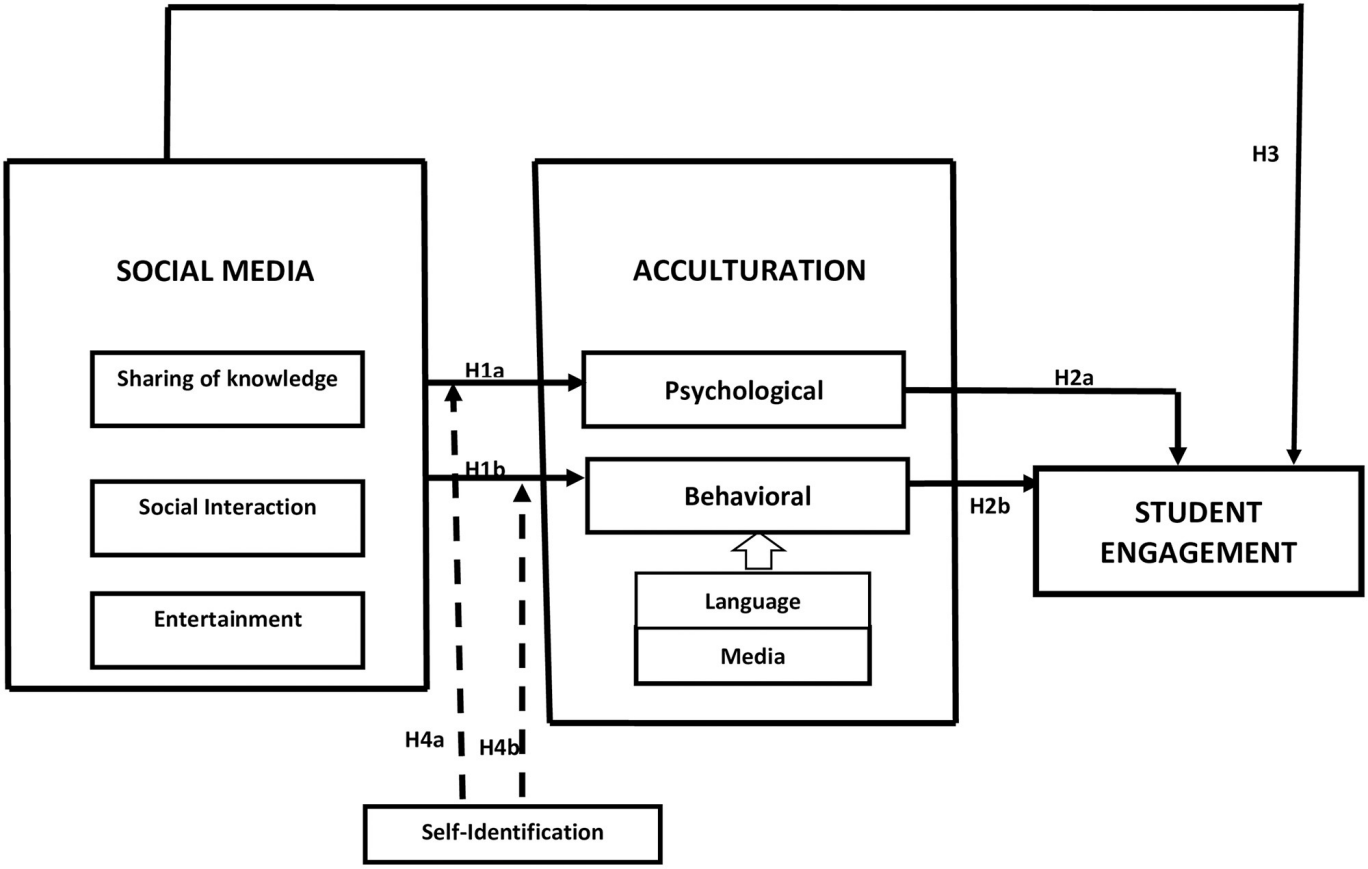
Hypothetical framework showing the relationship between social media, acculturation and student engagement.

In contrast to previous studies, this study focuses on how international students in China use social media content to enhance their acculturation process. According to [25], people use social media to seek information to fill knowledge gaps, fulfil entertainment needs, and connect with others. This research, therefore, aims to ascertain how international students use social media during their studies in Chinese universities, focusing on knowledge sharing, interaction, and entertainment. Bearing this in mind, we assume that when international students use social media to interact with their peers and family, enjoy various entertainment activities, and share knowledge across multiple channels, they are much more likely to understand the significance of what is happening in the host culture, maintain up-to-date knowledge, and reduce culture shock [20, 26]. Therefore, hypothesize that:

**H1a**. *There is a positive relationship between the use of social media by international students in Chinese universities and their mental acculturation to the host culture (see* Fig 2*)*.

**H1b**. *There is a positive relationship between the use of social media by international students in Chinese universities and their behavioural acculturation to the host culture (see* Fig 2*)*.

### 2.2 Acculturation and international students’ engagement

Students who move to a new country encounter various challenges, including adjusting to new cultural, ethnic, and academic surroundings [15]. Individuals attempting to re-establish themselves in a new setting are frequently driven to make adjustments that have mental and behavioural ramifications, making the transfer into the new environment extremely difficult [27]. International students come from different educational cultures and environments, and they may face a wide range of academic challenges and other forms of challenges such as the language barrier [28]. These challenges do not make it any easier for international students to adjust to a different educational setting [4].

Previous studies have examined the link between international students’ acculturation to their host culture and their engagement in school activities [29]. For instance, [30] demonstrate that international students have less social support, adopt abnormal coping strategies, and have greater inconsistencies between their academic expectations and university life experiences than their domestic counterparts. [31] evaluate the academic achievements of both local and international students in five Netherlands schools and find that international students outperform local students. Using primary data gathered from 383 research students in various universities in eastern China, [32] demonstrate a positive correlation between social media usage and student engagement. [33] used 1050 international student nurses from three different countries to demonstrate that using social media is critical to improving the learning of international student nurses. As a result, they propose that social media be used as a learning tool to make nursing curricula more inclusive and equitable.

Although these and other studies have demonstrated that the results are mixed and complex, their research focuses on either mental/psychological or behavioural changes during the acculturation process [31]. According to [17], acculturation occurs when people from different cultural backgrounds regularly interact, resulting in psychological and behavioural changes. These two acculturation processes are critical for international student engagement, but most previous research has focused on the psychological perspective [19].

This knowledge gap necessitates additional research into the impact of social media on the acculturation of international students from both a psychological/mental and behavioural standpoint. Therefore, following [4]., this paper contributes to the body of literature by investigating the impact of international students’ acculturation on student engagement (in our case, international students in China) from both psychological/mental and behavioural standpoints. We suggest that international students in China with a better knack for adapting to the host country’s culture (both mentally and behaviorally) may have higher levels of student engagement. Therefore, the study proposes that:

**H2a**. *There is a positive relationship between international students’ psychological/mental acculturation to the host culture and student engagement (see* Fig 2*)*.
**H2b**. *There is a positive relationship between international students’ behavioural acculturation to the host culture and student engagement (see* Fig 2*)*.

### 2.3 Social media use and international students’ engagement

Using social media in the classroom opens up several possibilities, including increased student engagement, the development of students’ social media skills, and the formation of students’ professional and peer networks [34]. Given the various challenges that international students may face, such as adjusting to a new educational and cultural environment, social relationships can aid in this process [35]. To this end, research on international students’ use of social media shows how emerging digital platforms can aid in their adaptation to new educational environments. According to [36], social media facilitates productive learning, networking, communication, and knowledge exchange. Similarly, [37],discover a positive relationship between social media and student engagement in the United States. [38], also show that most international students who spend more time on social media platforms feel more connected to their classmates than those who do not, thereby increasing academic collaboration. [39], noted that social networking sites significantly increased cooperation between domestic and international students, resulting in increased international student engagement. In a recent study, [34] find that incorporating social media into undergraduate and graduate courses helps increase student engagement and build peer and practitioner networks by showing students how course content relates to the real world and providing opportunities for them to connect with one another.

The use of social media to foster collaboration may raise concerns about privacy and security [40]. Given that sometimes there is no face-to-face contact and the opportunity to approach other students physically, international students may be at risk when working with people they do not know [41]. Furthermore, social media exposes international students to a wealth of data and information that might be difficult to digest and judge for accuracy owing to cultural differences [42]. Exposure to inappropriate online content, online stalking, and abuse all contribute to the inherent difficulties of using the social media of the host country [43]. Other studies have also reported that social media may distract students from engaging in school activities. Therefore, these issues and concerns overshadow the debate about whether social media can increase student engagement. However, because our focus is on postgraduate students who are older and more mature and thus better able to manage the risks associated with social media, we hypothesize:

**H3**. *There is a positive relationship between the use of social media by international students in Chinese universities and their engagement in education in the host country (see* Fig 2*)*.

### 2.4 Moderating role of ethnic self-identification

According to the identity theory, individuals are identified with a particular social group [44]. This social group is a group of people with a common identity. People find out which social groups they are a part of through the social group identification process, which helps them form their identities as members of an in-group or an out-group. Culture is one of the foundations for the identity of an individual [45]. Attempting to strike a balance between two different cultures or fitting in with the host culture will alter a person’s cultural identity. To form a new cultural identity, international students must go through a constant and dynamic process of negotiating between host and home cultures, i.e., maintaining the culture of their native country while also adapting to the culture of the country in which they reside [30]. International students with strong cultural identities are expected to have more difficulty adapting to the host country’s culture [46]. Ethnic self-identification highlights how migrants show and maintain their cultural characteristics when abroad [47]. Being highly motivated to make use of home media and communicate with people from the same country, especially speaking their local language, international students are considered unwilling to integrate into the host country which hinders their acculturation process [4]. But, according to [48] international students may exhibit dual cultural identities, which suggests that individuals can adopt the host country’s culture while remaining loyal to their own traditional culture. [49] proposed the coexisting cultural self-identification model. In another study, [50] concluded that international students who love their cultural identities and who regularly associate with those from the same ethnic heritage are more self-esteem. Therefore, we hypothesize that:

**H4a**. *International students’ self-identification has a moderating effect on the link between the use of social media and psychological/mental acculturation (see* Fig 2*)*.
**H4b**. *International students’ self-identification has a moderating effect on the link between the use of social media and behavioural acculturation to the host culture (see* Fig 2*)*.

## 3. Method

### 3.1. Ethical approval

Ethical approval was sought from the Jiangsu University Institutional Review Board (JUIRB/SEM/2022/97) and the International Education Unit (IEU) in China to conduct the study. A recording device was used to take oral consent from participants as indicated by the ethics committee. The purpose of the study and other details were disclosed to the authorities and participants. Participants were not financially induced or coerced to take part in the study. It was explained to them that their participation was voluntary.

### 3.2. Participants and materials

To test the hypotheses stated above, a purposive sampling approach was used to select postgraduate international students in China who were 18 years of age or older and had studied in a Chinese university for more than six months. We conducted online surveys by administering a questionnaire to gather responses from international students in several universities across China. The research is quite interesting because the survey was administered according to the research plan and methodology. The investigation proceeded without deviating from the chosen methods of analysis towards achieving the research objectives. For instance, to get adequate and first-hand information to support the research findings and make a generalization on social media usage, survey research was chosen over other research designs. This gives room for more generalized results and outcomes in situational analysis. We also advised everyone qualified to partake in the research to spread the word by inviting their friends and colleagues to join them. Participants were asked to provide accurate information and were guaranteed that their data would be collected without revealing any personal information and would only be used for this study. Meanwhile, participants were asked to stop participating in the online questionnaire if they had any hesitations about it.

The SEM method is appropriate for establishing a correlation among variables, assessing the theoretical model and getting the research hypotheses tested with firm results. More so, the chosen method offers immediate and long-term results. The research model was tested in a pilot study on a convenience sample of forty social media users drawn from the studied population to ensure its suitability, reliability, validity and freedom from error. Following the validation procedure, a total of 395 responses were collected using online questionnaires in the English language over three months from 32 different universities located across mainland China. These universities were selected because they have sufficient numbers of international students, who enroll in English language programmes. However, 41 responses were removed from the dataset due to uncompleted data. We did this to get a complete dataset for the analysis. Besides, in dealing with missing data at random, related data can be deleted to reduce bias. Especially in cases where the data gathered are more than enough for generalizing the subject under study.

### 3.3 The measures considered in the survey

The questionnaire used for the study is made up of two parts. The first portion included questions about the participants’ demographics (gender and age). The second part contains measures, which were built on earlier studies.

#### i. Demographic variables

Participants were instructed to state their (i) gender (male or female), (ii) age (on an average-point Likert scale), ranging from 1 (18 years–25years) to 5 (41years and above) and (iii) the number of residences in China on average-point Likert scale, ranging from 1 (less than six months) to 4 (more than three years).

#### ii. Social media usage

In the same way as [4] measured social media usage, we measured it using three constructs: information sharing, interpersonal interaction, and entertainment. The participants were asked to indicate whether or not they believe that using social media platforms improves their ability to share knowledge, allows them to interact with their peers, and enables them to entertain themselves. Each statement was gauged according to the average-point Likert scale, ranging from (1) strongly disagree to (5) strongly agree. Sample statements related to knowledge sharing, entertainment and social interaction are provided in Table 3. Though the result regarding the hypothesis of ‘usage’ seems quite broad, the usage component of social media analyzed in the study can further be explored in other studies to complement the literature on research and development under the subject under discussion.

#### iii. Acculturation

Acculturation was also assessed using two constructs: Psychological/mental and behavioural acculturation, both of which were used to measure it as in [4]. Sample questions for psychological variables include: “With which group of people do you feel you share most of your beliefs and values?”, “With which group of people do you feel you have the most in common?” etc. The variable ’behavioural’ was also developed based on earlier conceptual frameworks, mainly those outlined by [19]. Like in [19] and [4], we consider behavioural variables under two frameworks, i.e. language and media use. Sample terms include: “I feel very comfortable speaking my local dialect with my friends”–for language and “The Internet websites that I browse are mostly in Chinese”–for media (See Table 3 for details). Each statement was scored on a 5-point Likert scale between 1 (strongly disagree) and 5 (strongly agree)

#### iv. Self-identification

Self-identification is a feeling of belonging and devotion to a particular ethnic group. In other words, it involves being associated with a specific ethnic group that shares common values and attitudes and sentiments of belonging and commitment. This variable was developed based on earlier conceptual frameworks, mainly those outlined by [51]. On this note, we measure this variable using four constructs. See details in Table 3.

#### iv. Student engagement

[52] describe student engagement as “participation in educationally effective practices, both inside and outside the classroom, which leads to a range of measurable outcomes”. Based on this definition, like in [32], we adopt the student engagement measure from [53]. Sample terms include: “I often miss my school/research lab”, “I often cut/skip my classes/research workshops”, etc. See Table 3 for details. Each statement was scored on a 5-point Likert scale between 1 (strongly disagree) and 5 (strongly agree).

#### v. Language

Research by [54] confirmed that the utilization of social media has been significantly perceived to have positively impacted learning the English language in terms of writing style, reading skills, listening and lexical variation, communication skills and grammar usage. In the future other potential underlying variables other than what we have tested could be factored into the analysis of student usage of WeChat and other social media platforms that is tied to understanding the language before its effective usage. Because having a prior understanding of language makes it easier to use other social media platforms for timely purposes with acculturation and easier time engaging in the coursework of study among students.

## 4. Data analysis

In the first section, we present the descriptive statistics which offer insight into the general characteristics of the respondents (see Table 1). Subsequently, a correlation matrix was executed to investigate relations amongst the main measures (See Table 2). In the second section, we further examine the correlation outcome using structural equation modelling (SEM) to assess the above theoretical framework of the study and the proposed hypotheses. As a result, in this second stage, we first check the measurement model using the Amos 24, which was then used to find the causal relations between the observed and unobserved construct (see Table 3). The model was evaluated by looking at its reliability and validity testing results, among other things. In the second step, a SEM is evaluated using a multiple regress technique like hypothetical relationships based on the sign, magnitude, and significance level (see Table 4).

**Table 1:** Demographic information on international students in China.

Items	Frequency	Percentage
**Gender**		
**Male**	272	77.0
**Female**	82	23.0
**Age**		
**18–25**	12	3.39
**26–30**	124	35.03
**31–35**	140	39.55
**36–40**	72	20.34
**41 and above**	6	1.69
**Residence length**		
**Less than 1 year**	42	11.84
**Between 1 to 2 years**	45	12.71
**Between 3 to 4 years**	236	66.67
**5 and above**	31	8.76
**Education level**		
**Masters level**	248	70.06
**PhD level**	106	29.94
**List of social media used**		
**WeChat**	221	62.43
**WhatsApp**	86	24.29
**Facebook**	36	10.17
**Instagram**	11	3.11
**Using social media duration**		
**Less than 30 mins per day**	20	5.65
**Between 30 mins and 1 hour per day**	43	12.15
**Between 1 to 2 hours per day**	71	20.06
**Between 2 to 5 hours per day**	96	27.19
**Between 6 to 8 hours per day**	34	9.60
**More than 8 hours**	90	25.42

**Table 2:** Correlation matrix of all variables.

	**KS**	**EM**	**SOI**	**PS**	**LAN**	**MED**	**SI**	**SE**
**1. Knowledge sharing (KS)**	0.883							
**2. Entertainment (EM)**	0.273	0.884						
**3. Social interaction (SOI)**	0.199	0.211	0.823					
**4. Psychological (PS)**	0.344	0.321	0.432	0.910				
**5. Language (LAN)**	0.234	0.421	0.344	0.341	0.904			
**6. Media (ME)**	0.132	0.322	0.311	0.234	0.315	0.890		
**7. Self-identification (SI)**	0.321	0.342	0.391	0.123	0.134	0.385	0.880	
**8. Student engagement (SE)**	0.345	0.221	0.211	0.133	0.284	0.113	0.412	0.929

Note: The diagonal elements (in bold) are the square root of average variance extracted (AVE) whereas the other entries reflect the correlations.

**Table 3:** Results of the items of construct, factor loadings and reliabilities.

Variables	Items		Loadings	CA	CR	AVE
**Social Media Usage**						
• **Knowledge sharing**	The advice I receive from other members using the social media allows me to conduct tasks more successfully.	KS1	0.846	0.954	0.921	0.693
	The advice I receive from other members using the social media has increased my understanding.	KS2	0.867			
	The advice I received from other members using the social media has increased my knowledge.	KS3	0.768			
• **Entertainment**	I use social media as a source of entertainment.	ENT1	0.794	0.864	0.831	0.783
	I use social media to play games.	ENT2	0.946			
	I use social media to listen to music	ENT3	0.924			
	Chatting on social media with my friends is very entertaining.	ENT4	0.732			
• **Social interaction**	I use social media to interact with others	SOC1	0.822	0.754	0.741	0.678
	I use social media to interact with my friends.	SOC2	0.963			
**Acculturation**						
• **Psychological**	With which group(s) of people do you feel you share most of your beliefs and values?	PSYC1	0.863	0.986	0.863	0.838
	With which group(s) of people do you feel you have the most in common?	PSYC2	0.735			
	With which group(s) of people do you feel the most comfortable?	PSYC3	0.872			
	In your opinion, which group(s) of people best understands your ideas (your way of thinking)?	PSYC4	0.957			
	Which culture(s) do you feel proud to be a part of?	PSYC5	0.911			
	In which culture(s) do you know how things are done and feel that you can do them easily?	PSYC6	0.863			
	In which culture(s) do you feel confident that you know how to act?	PSYC7	0.756			
	In your opinion, which group(s) of people do you understand best?	PSYC8	0.852			
	In which culture(s) do you know what is expected of a person in various situations?	PSYC9	0.856			
	Which culture(s) do you know the most about the history, traditions, and customs, and so forth?	PSYC10	0.976			
• **Behavioral**						
*a) **Language***	I feel very comfortable speaking my local dialect with my friends.	LANG1	0.964	0.947	0.865	0.818
	Many of my favorite shows on TV are in Chinese	LANG2	0.739			
	I like to read books or magazines in Chinese	LANG3	0.867			
	I always watch movies in Chinese	LANG4	0.764			
*b) **Media***	The Internet websites that I browse are mostly in Chinese.	MEDIA1	0.862	0.894	0.772	0.792
	I enjoy listening and singing Chinese songs much more than songs in other languages.	MEDIA2	0.962			
	My favorite actors/actress are from my country	MEDIA3	0.812			
**Self-identification**	I am very attached to all aspects of my culture	SELF1	0.728	0.916	0.873	0.776
	My culture has had the most positive impact on my life	SELF2	0.975			
	I consider my culture to be rich and precious	SELF3	0.883			
	The people that I admire the most are mostly those from my culture	SELF4	0.982			
**Student engagement**	I often miss my school/research lab	SE1	0.839	0.861	0.785	0.864
	I often cut/skip my classes/research workshops	SE2	0.875			
	I often get late to reach my school/research lab	SE3	0.985			
	I often go to my school/research lab without prepared	SE4	0.855			
	I often go to my school/research lab without research tools such as needed research apparatus/laptop	SE5	0.792			
	I often go to my school/research lab without needed books/material etc	SE6	0.898			

**Note**: CA represents Cronbach alpha; CR is the composite reliability; and AVE denotes the average variance extracted.

**Table 4:** Hypotheses testing.

Hypotheses Path			β	S. E	CR		P	
**H1a**	Social media use → Psychological/mental acculturation		0.834	0.163	6.245		***	Accepted
**H1b**	Social media use → Behavioral acculturation		0.982	0.182	5.321		***	Accepted
**H2a**	Psychological acculturation → Student engagement		0.195	0.134	4.835		***	Accepted
**H2b**	Behavioral acculturation → Student engagement		1.232	0.175	5.423		***	Accepted
**H3**	Social media use → student engagement		0.634	0.042	6.213		***	Accepted
	Moderation effect (self-identification)			Unstandard Coefficient	Standard Coefficient	t	Sig.	
			β	Std. error	Beta			
**H4a**	Social media use → Psychological acculturation	(Constant)	2.434	0.432		6.373	0.000	Accepted
		Social media	0.323	0.042	0.239	3.946	0.003	
		Psychological acculturation	0.163	0.062	0.174	2.707	0.000	
**H4b**	Social media use → Behavioral acculturation	(Constant)	1.052	0.212		5.167	0.000	Accepted
		Social media	0.632	0.049	0.537	8.334	0.001	
		Behavioral acculturation	0.223	0.043	0.262	7.616	0.000	

### 4.1 Stage one: Descriptive statistics

Of the final 354 international students, 82 were female students (23%) and 272 were male students (77%). Concerning age, 3.39% of the participants were of the age bracket 18–25 years, 35.03% were of the age bracket 26–30 years old, 39.54% were between 31 and 35 years old, 20.34% were between 36 and 40 years old and 1.70% between 41 and 45 years old. Also, of the total number of international students, the majority of them had been living in China for between 3 to 4 years (66.67%), 11.86% between 1 and 2 years, and 8.76% have been in China for 5 years and above. 70.06% were pursuing their master’s degree and 29.94% their doctorate. According to the survey, the most highly used social media is WeChat (62.43%), followed by WhatsApp (24.29%), Facebook (10.17%) and Instagram (3.11%). These social media is used between 2 and 5h per day (27.12%) and more than 8 hours (25.42%).

### 4.2 Correlations among variables

Table 2.

### 4.3 Stage two (a): Measurement model results

As previously stated, the current study used SEM and AMOS 24 software to assess the theoretical model and research hypotheses. This process is carried out in two phases: In the first phase, a confirmatory factor analysis (CFA) was performed to test the measurement model. In the second phase, we assessed the hypotheses of the theoretical model employing multiple regression techniques. SEM was selected because it makes it easier to assess hypotheses based on empirical measures that propose complex links outlined in theoretical models.

In the first phase, we carried out a confirmatory factor analysis (CFA). CFA is a statistical method for confirming the factor structure of a group of observed variables. It aids in the identification and determination of construct validity and reliability (that is, the convergent, discriminant, and validity). The total model was subjected to CFA using AMOS version 24.

A reliability measure is applied in examining internal consistency by computing observed items and avoiding superfluous dimensions generated by factor analysis owing to garbage items [15]. The coefficient alpha (CA) was utilized to test the internal consistency reliability because it is the most generally used internal consistency technique that reveals how distinct items can measure diverse aspects of a construct [26]. The CA scale runs from zero to one, with values below 0.6 indicating low reliability [55]. [56] stated that when internal consistency is low, the content of the items will be too heterogeneous. CA should have a minimum threshold of 0.7 [57]. The CA in this research was greater than 0.70 (see Table 3), indicating that the findings are acceptable because of past studies see [57].. The construct-level reliability, also known as composite reliability (CR) demonstrated that items belonging to the same constructions had a strong association. It was suggested that the composite reliability be larger than 0.7 [57]. Similarly, in the present study, the CR were above 0.70 indicating that the results are acceptable in light of the previous research (see Table 3).

In this study, the discriminant and convergent validity tests were carried out to validate the measure. Discriminant validity relates to how much measures differ from other operationalizations, demonstrating that the construct is genuinely different from other constructs [58]. Another technique for checking discriminant validity is by computing the extracted average variance for the constructs and comparing it to the square correlation between them [51]. [56] recommended that values for each construct be larger than 0.50 when calculating the average variance extracted (AVE). The results for the AVE in this study were above 0.50 ranging 0.678 from to 0.838, supporting the discriminant validity. An alternative measure to confirm the evidence for discriminant validity is when estimated correlations among factors were lower than the required value of 0.92 [4]. Convergent validity was investigated based on construct reliabilities and was used to test the construct’s homogeneity. The correlation matrix for constructs with a cut-off value of 0.92 is shown in Table 2.

The Mann-Whitney U test was used to calculate possible non-response bias by comparing the differences between early and late responders about the average of all variables [55, 59]. Here, the first fifty observations were considered early responders, while the final fifty were considered late responders, based on the proportions of the times at which the online questionnaires were answered and returned. The results revealed there is no statistically significant difference between early and late responders; thus, non-response bias is not an issue.

We used the Harman single-factor test to assess the common latent factor and common method bias suggested by earlier research, using differences in chi-square values among the original (or initial) and fully constrained model [4, 46, 55]. The findings suggest that the two models are statistically different and share a variance. The model’s initial results were assessed without considering the method biases. Furthermore, we adopt the [60] four common method variance (CMV) categorization sources. As a result, measuring context effects played a large role in the magnitude of CMV in our investigation. The model’s original results were then examined without considering the method biases, and CFA was suggested. The model fit was appraised using the Comparative Fit Index (CFI), with a value of 0.932 greater than 0.90 indicating an excellent fit and for the Root Mean Square Error of Approximation (RMSEA), a value of 0.075 less than 0.08 indicates an acceptable fit. The Tucker-Lewis index (TLI), incremental fit index (IFI), and normed fit index (NFI) of the suggested operational model were 0.915, 0.912 and 0.882, respectively, with a chi-square of 2442.285 (degrees of freedom, df = 735; p.001). Furthermore, the findings reveal that CMV was not the primary cause of the changes in the observed items [61].

Having done all these, in the second phase, we carried out the hypotheses testing of our model.

### 4.4 Stage two (b): Hypotheses testing

In this section, we tested our hypothesized model. To test the model fit, a variety of goodness-of-fit measures were used and this includes, including the comparative fit index (CFI), the Chi-square statistic, the root mean square error of approximation (RMSEA), and the standardized root mean square residual (SRMR). An insignificant Chi-square, an RMSEA value of less than 0.06, a CFI value above 0.95, and an SRMR below 0.05 are all characteristics of a good research model.

The model fits the data based on a variety of goodness-of-fit measures with a chi-square of 2324.342 (degrees of freedom, df = 645; p < .000), RMSEA = 0.032, SRMR = 0.036 and CFI = 0.953. Table 4 shows the results from the model. The H1a and H1b hypotheses, which propose a direct connection between social media use and mental and behavioural outcomes, respectively, suggest that students’ use of social media is positively related to students’ psychological/mental acculturation (β = 0.834, t = 6.245, and p = 0.000) and behavioural acculturation (β = 0.982, t = 5.321, and p = 0.000). For research hypothesis H2a, the findings indicate that there is a significant linkage between psychological/mental acculturation and students’ engagement (β = 0.195, t = 4.835, and p = 0.001). Similarly, H2b shows a statistically significant link between behavioural acculturation and students’ engagement (β = 1.232, t = 5.423, p = 0.000). The results for hypothesis H3 show that social media usage is positively linked with students’ engagement (β = 0.634, t = 6.213, p = 0.000). Finally, the outcomes reveal that self-identification is a mediator in the relationship between social media use and mental acculturation (H4a) and behavioural acculturation (H4b). In other words, the outcomes suggest that the effect of social media on behavioural and psychological/mental acculturation could increase due to self-identification. The results of the regression coefficient are also illustrated in Table 4.

## 5. Discussion and implications

### 5.1 Discussions

This research paper is one of the first to investigate the linkages between international student’s use of social media and the impact it has on their acculturation and participation in school activities while in China. Specifically, we discovered that international students use of social media is positively linked with their acculturation which supports H1a and H1b. In other words, the study’s findings indicate that when international students use social media to share or exchange information, create contact, or entertainment, they are more likely to become mentally and behaviorally acclimatized to the host culture throughout their time studying in China.

Researchers have previously discovered that this particular group of students may experience greater challenges with language and communication as well as sentiments of homesickness and loneliness than the general student body [24]. However, given the widespread use of social media, this research reveals that international students may obtain the majority of their knowledge from online media platforms. According to our findings, international students in China spend anywhere between one and six hours every day on social media. They primarily use WeChat (about 62%), followed by WhatsApp (24%). This is consistent with previous findings that the amount to which international students rely on WeChat appears to contribute to their overall contentment with Chinese living [14]. A large number of previous research have demonstrated that the more an international student interacts with the host country’s social media, the more likely he or she is to acclimatize to the host culture [6]. As a result of this finding, international students may use Chinese social media such as WeChat to communicate with local communities and students in their host countries.

The research findings also reveal that there is a connection between international students’ psychological/mental and behavioural acculturation to Chinese culture and their participation in school activities (i.e. H2a and H2b). This suggests that when overseas students are acculturated to the host culture from a psychological standpoint, their self-esteem is boosted, which leads to an increase in their participation in school activities, hence boosting their student engagement and overall academic success. Similarly, from a behavioural standpoint, international students who utilize social media are more comfortable connecting with people from the host country, which may make it simpler for them to participate in school activities and, as a result, increase their level of student engagement. The use of social networking sites such as WeChat from the host nation may also be connected with an improved comprehension of lecture content and involvement in school activities thereby improving student engagement. It is in line with the findings of other experiential research, such as [32] which observed a positive connotation between acculturation and student engagement in a university setting.

Regarding hypothesis (H3), the study also discovers that there is a direct positive link between students’ use of social media and their engagement in school activities. Student engagement has been recognized as one of the most essential markers for assessing the quality of an international educational experience. According to our findings, the use of social media can encourage international students to become more involved in their academics and extracurricular activities. These findings are congruent with the findings of previous studies conducted in different circumstances. Example: According to [32], social media improves students’ communication, cooperation, and relationship-building skills when they interact with others. [4] asserted that the online setting offers access to a variety of resources and educational tools that can help international students learn more effectively and efficiently.

We also found that self-identification (which is represented by H4a and H4b) plays a moderating role in the connection between international students’ use of social media and their acculturation. This is notable since some earlier study has seen acculturation as a one-dimensional process. This emphasizes the notion that overseas students are less likely to adopt another country’s media or connect with those from another culture despite their deep connection to their culture [35]. This suggests that having a strong ethnic identification does not inevitably result in less acclimation to another culture and therefore, people can be caught between two different cultures without sacrificing their cultural identities supporting the bi-dimensional acculturation [62]. These findings support [63]. conclusion that self-identification with the host country has little bearing on psychological well-being. Instead, sojourners’ self-esteem can be boosted by maintaining and practising their cultural identity. This implies that when international students are comfortable with their own identity and culture, they are less concerned about cultural differences and tend to respect other cultures. Similar results are presented by [4] who observed that Chinese students in the United States with strong ethnic identities and who frequently communicate with family or friends from their ethnic background appear to combine excellent academic accomplishment with low stress. We believe that the findings of this research add to the four-acculturation framework proposed by [17]. (that is, assimilation, integration, separation, and marginalization), which emphasizes the importance of cultural balancing and the coexistence of two or more cultures in society [64].

### 5.2 Theoretical and practical implications

Theoretically, this study broadens the scope of literature on acculturation and social media by providing a more in-depth picture of the relationship between various types of acculturation processes (from psychological/mental and behavioural perspectives), social media, and student engagement. Most prior research has examined the relationship between social media use and the acculturation stress faced by international students during their transition to a new culture from a psychological standpoint. The study could contribute to acculturation theory because it examines the possible effects of social media use on the process of acculturation for international students in China, both psychologically and behaviorally. The findings of this study highlight the significant role that using social media plays among international students, as well as its ultimate impact on the students’ acculturation process and academic performance while abroad.

Practically, our results are quite relevant in the field of social media and higher education. This research sheds light on the experiences of international students (in our case, international students in China) and will assist school administrators in developing a solid university-student connection that will benefit both parties in the long run. Even while some studies have suggested that using social media to learn about a new culture may hinder immigrants’ ability to acculturate to the new culture, our data reveal that the opposite is true among international students in China who use social media to learn about their new surroundings. University officials should not be concerned that international students with strong ethnic identities will struggle to integrate into the host culture. Instead, they should increase their investments and support these students. These investments and forms of support can include: (i) giving international students a welcome package with stationery, gift cards to a local store, and a sim card so they can use their phone; (ii) using visual aids when teaching; (iii) encouraging them to use examples from their own cultures in classroom discussions; and (iv) provide them full support in language learning and event planning to foster interactions between international and indigenous students.

Aside from that, there is a positive association between the use of social media by overseas students and their ability to participate in school-related activities. It is hoped that these findings would assist educational institutions, lecturers, and research supervisors to better recognize the benefits related to using social media to increase student participation in school activities. To put it another way, our study reveals that social media can be quite beneficial for international students in several ways. For example, social media can enable overseas students to collaborate more effectively with persons in the host country who share their research interests, thereby increasing their chances of participation in academia. In addition, the more connections international students made with people in the host country may result in greater information sharing. This information sharing may then result in increased participation in school activities.

On a more general note, international students require programs that enable them to better integrate with students from the host culture, reducing the negative impacts of acculturative stress. Therefore, the following strategies and actions can be implemented. First, it is recommended that school educators ask their school administration for specialized materials and training to aid in the acculturation of international students. Second, some ethno-specific leisure activities are needed to better cater to people who are still in the acculturation process and those who do not wish to become acculturated. Faculty can help by clarifying classroom and academic standards through class discussions and course information and encouraging an open and tolerant classroom environment to embrace international student diversity. Third, universities should provide cultural programs like cultural tours, taichi (a Chinese martial art), and a learning atmosphere that allows students to adapt to the university and Chinese culture through various experiences. International students should also have the opportunity to participate in voluntary social programmes and activities with the local Chinese community, like cultural sharing, counselling, and recreational activities where they can study Chinese culture with the Chinese, as it is an effective way to get social support from Chinese people. Fourth, by actively fostering the creation of broader social networks involving a wider range of nationalities, school counsellors or authorities can aid in the acculturation of international students. This could include assisting students in developing extra social skills and providing information on how to meet new individuals on campus, not just in the classroom but also outside the campus. Finally, employing teachers and school administrators from different countries may help promote international students’ acculturation. These teachers may provide inspiration and support to new students who are still in the acculturation process.

## 6. Conclusion, limitations and future studies

There are several studies investigating the connection between social media, acculturation process and student engagement. However, most of these studies focus on Chinese students’ experiences in UK and American universities and how their expectations were met or not. Given that recently China is increasingly becoming a recipient of international students to boost inward student mobility, in this research, we focus on international students in China by examining the influence of the use of modern social media on international students’ acculturation and their engagement in school activities. But more importantly, the findings of the paper established the tentative arguments before the study revealed the following outcomes; (1) direct connection between social media use and mental and behavioural outcomes, respectively, suggests that students’ use of social media is positively related to students’ psychological/mental acculturation. (2) There is a significant linkage between psychological/mental acculturation and students’ engagement. (3) There is a significant link between behavioural acculturation and student engagement. The results for the hypothesis show that social media usage is positively linked with students’ engagement. In our regression analysis, self-identification mediates the relationship between social media use and mental acculturation and behavioural acculturation. In other words, the outcomes suggest that the effect of social media on behavioural and psychological/mental acculturation could increase due to self-identification. The outcomes of this study are particularly useful for schools that host a large number of international students and that have a multi-national recruitment strategy. The findings of the study demonstrate the critical role played by social media among international students, as well as the ultimate impact it has on their studies.

The study has several flaws which can be explored in future studies. First, though social media use is an important predictor of acculturation, this study did not investigate the influence of demographic variables like age, sex, personal traits, university program, and years of sojourning on acculturation. Another problem is that the participants’ gender ratios were not evenly distributed. There were more males than females which means future research is should try to even the balance between gender ratios. Furthermore, because the survey only included a small number of international students in China, the study’s sample may not be a true representation of the sample of the overall population of international students in China. Another issue is that the study treated all of the international students as if they were from the same country and culture, even though they came from diverse countries and cultures. In other words, the current study was unable to distinguish between participants’ cultural backgrounds as a factor that influences the study’s outcomes. It would have been preferable to divide the participants into groups based on their country of origin before conducting the analysis. The findings inspire future studies to recruit a larger number of participants while also taking into consideration the aforementioned issue to re-examine the effect of social media on acculturation and student engagement in the analyses.

## Supporting information

S1 Dataset (DOCX)
