# Supplementary material for: The impact of social media use on student engagement and acculturative stress among international students in China
Source: PLoS One. 2023 Apr 14;18(4):e0284185. doi: 10.1371/journal.pone.0284185 (PMC10104301; doi:10.1371/journal.pone.0284185)
Supplement: S1 Dataset — (DOCX) [file pone.0284185.s001.docx]

FROM THE JOURNAL

2. We note your Data Availability statement as follows:

"The shared link and Don to the data is below:

Doi:10.17632/c27jz8y4wx.2

<https://data.mendeley.com/datasets/c27jz8y4wx/draft?a=0f24a98f-9867-4eb1-a550-5f3d2d0cb5c2> "

We note that the Doi 10.17632/c27jz8y4wx.2 does not appear to currently be active. Before we are able to proceed to publication, we request that you ensure that the dataset at the provided DOI has been made publicly available, and that your Data Availability statement is accurate and correct for publication.

**Author Response**

This is the published link and DOI to the data repository site.

<https://data.mendeley.com/datasets/c27jz8y4wx/1>

DOI:[10.17632/c27jz8y4wx.1](http://doi.org/10.17632/c27jz8y4wx.1)

Other ways to also see it.

Find Data link <https://data.mendeley.com/research-data/> after clicking that you enter the topic, and it will clearly show up.

<http://dx.doi.org/10.17632/c27jz8y4wx.1>

Bibliographic Details

DOI:[10.17632/c27jz8y4wx.1](http://doi.org/10.17632/c27jz8y4wx.1)

URL ID <https://data.mendeley.com/datasets/c27jz8y4wx>;

<http://dx.doi.org/10.17632/c27jz8y4wx.1>;

<https://dx.doi.org/10.17632/c27jz8y4wx.1>;

<https://data.mendeley.com/datasets/c27jz8y4wx/1>

Below are the images of the email from the digital commons data set which indicates that it has been published

Content of the email

## Your dataset has been published

Dear Blessing Dwumah Manu, 

Thank you for submitting your dataset, it has now been published on the repository. You can view it here: <https://data.mendeley.com/datasets/c27jz8y4wx/1>

Thanks,
The Digital Commons Data team
